# Influence of Amitraz and Oxalic Acid on the Cuticle Proteolytic System of *Apis mellifera* L. Workers

**DOI:** 10.3390/insects3030821

**Published:** 2012-08-27

**Authors:** Aneta Strachecka, Jerzy Paleolog, Krzysztof Olszewski, Grzegorz Borsuk

**Affiliations:** Laboratory of Experimental and Environmental Biology, Department of Biological Basis of Animal Production, University of Life Sciences in Lublin, ul. Akademicka 13, PL-20-950 Lublin, Poland; E-Mails: aneta.strachecka@up.lublin.pl (A.S.); krzysztof.olszewski@up.lublin.pl (K.O.); grzegorz.borsuk@up.lublin.pl (G.B.)

**Keywords:** proteases, protease inhibitors, *Apis mellifera*, bees, cuticle, amitraz, oxalic acid

## Abstract

This work verifies that amitraz and oxalic acid treatment affect honeybee cuticle proteolytic enzymes (CPE). Three bee groups were monitored: oxalic acid treatment, amitraz treatment, control. Electrophoresis of hydrophilic and hydrophobic CPE was performed. Protease and protease inhibitor activities (*in vitro*) and antifungal/antibacterial efficiencies (*in vivo*), were analyzed. Amitraz and oxalic acid treatment reduced hydrophobic, but did not affect hydrophilic, protein concentrations and reduced both hydrophilic and hydrophobic body surface asparagine and serine protease activities in relation to most substrates and independently of pH. The activities of natural cuticle inhibitors of acidic, neutral, and alkaline proteases were suppressed as a result of the treatments, corresponding with reduced antifungal and antibacterial activity. Electrophoretic patterns of low-, medium-, and high-molecular-weight proteases and protease inhibitors were also affected by the treatments.

## 1. Introduction

Proteolytic enzymes (proteases and protease inhibitors) are active in extra- and intracellular proteolysis and take part in such processes as zymogene activation, the release of hormones and active proteins from their precursors, transport through the cell membranes, witin-cell complex protein processing and receptor activation [1,2,3,4,5]. Proteolytic enzymes are present in the honey-bee alimentary duct, hemolymph, moult liquid, and venom [1,3,6,7,8]. It has been recently shown that in *Apis mellifera*, many cuticle proteins display protease and protease inhibitor activities [9,10,11].

Colony collapse disorder (CCD) has recently inspired numerous studies [12,13,14]. For a better understanding of CCD etiology it is important to examine all mechanisms and conditions of apian resistance [15,16]. A significant component of the resistance is the layer of active cuticle surface proteins that protect the honeybee against pathogen invasion [9,10]. Therefore this proteolytic system may be also important for a better understanding of CCD etiology. To date, serine, cysteine, and asparagine proteases, as well as metalloproteases, have been found on the apian cuticles. The activity of the cuticle proteolytic system was higher in an unpolluted environment than in a polluted one [11]. Therefore, environmental factors may be of crucial importance to the apian body surface resistance.

The diseases’ complex caused by infestation of *Varroa destructor* in honeybees is a serious problem in contemporary apiculture [17] and is an etiologic factor of CCD. The situation worsened at the beginning of the current century. *V. destructor* was found to be entirely or partially resistant to some varroacids (miticides) [18,19,20,21], including amitraz [*N*-methylbis(2,4-xylyliminomethyl)amine]. Amitraz, an insecticide used to prevent tick and mite infestation of crops, humans, domestic animals and bees, is an α_2_-adrenergic receptor agonist that leads to paralysis and death in mites. 

Oxalic acid ([21,22,23], and review in [24]) has been promoted as a varroacid in response to the reduced effectiveness of other chemotherapeutics. However, bee resistance could decrease as a result of varroacid treatment [20]. Polish apiarists also report during their meeting that amitraz and oxalic acid may promote the development of other diseases, especially diseases caused by fungi (mycosis). This could be connected with the suppression of the body surface proteolytic system.

Therefore, in this research we studied how amitraz and oxalic acid, administered according to the common veterinarian recommendations, affected the activity of the cuticle proteolytic defense system in *A. mellifera* workers. Bees were examined just before, and two weeks after, the treatments.

## 2. Results and Discussion

Values of all the examined traits were similar in all the groups (differences between group-averages and group-effects were insignificant; *p* < 0.05) in the bee samples taken just before the treatment. Hence, the bees from all the groups were very similar and results are not presented here. 

**Figure 1:**
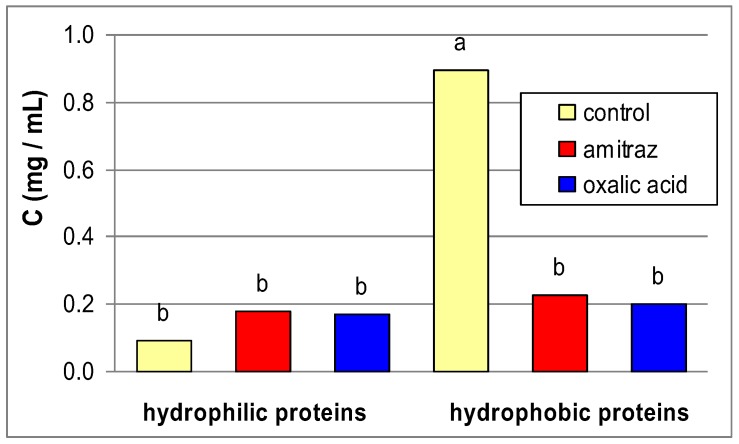
The influence of amitraz and oxalic acid on mean (n = 60) protein concentrations (**C**) in the cuticle samples of *A. mellifera* workers in comparison to the control. Key: various lowercase letters—the differences are significant for *p* ≤ 0.05, for comparisons made separately within hydrophobic and hydrophilic proteins (2 × ANOVAs and Duncan multiple range tests).

The amitraz and oxalic acid treatment reduced hydrophobic but did not affect hydrophilic protein concentration (Figure 1) on the apian cuticles. Amitraz and oxalic acid treatments reduced both hydrophilic and hydrophobic cuticle protease activities with most of the substrates and independently of the pH value (Table 1). 

**Table 1:** Proteolytic activities [U/mg] in relation to various substrates in the cuticle samples of *A. mellifera* workers treated with amitraz and oxalic acid, as compared with the control.

			Control	Amitraz	Oxalic acid
Substrate	proteases	pH	Mean ± se	Mean ± se	Mean ± se
Albumin	Hydrophilic (rinsed in water)	2.4	2.17 ± 0.77	4.74 ± 3.21	2.55 ± 1.79
		7	13.88 ± 6.82	8.63 ± 3.74	7.74 ± 3.42
		11.2	21.889^a^ ± 5.06	20.15^a^ ± 4.61	10.52^b^ ± 1.55
	Hydrophobic (rinsed in triton)	2.4	12.66^b^ ± 0.03	13.74^b^ ± 1.93	19.57^a^ ± 4.72
		7	7.867 ± 0.05	14.53 ± 1.85	20.13 ± 5.12
		11.2	167.71^a^ ± 0.22	13.91^b^ ± 1.77	19.54^c^ ± 4.70
hemoglobin	Hydrophilic (rinsed in water)	2.4	1.87 ± 0.87	1.00 ± 0.50	1.00 ± 1.00
		7	1.73 ± 0.16	1.08 ± 0.43	1.79 ± 1.54
		11.2	2.56^a^ ± 0.33	0.44^b^ ± 0.16	1.55^ab^ ± 0.83
	Hydrophobic(rinsed in triton)	2.4	15.58^a^ ± 0.14	5.67^b^ ± 0.99	2.87^c^ ± 0.71
		7	20.64^a^ ± 0.07	5.22^b^ ± 1.15	4.38^b^ ± 1.17
		11.2	10.72^a^ ± 0.07	2.96^b^ ± 1.23	3.29^b^ ± 0.72
cytochrome C	Hydrophilic (rinsed in water)	2.4	4.65^a^ ± 0.61	0.15^b^ ± 0.07	1.53^c^ ± 0.15
		7	7.42^a^ ± 0.82	0.93^b^ ± 0.11	0.68^b^ ± 0.25
		11.2	9.54^a^ ± 0.31	2.19^b^ ± 0.23	1.05^c^ ± 0.11
	Hydrophobic (rinsed in triton)	2.4	12.27^a^ ± 0.10	8.92^b^ ± 1.15	12.33^a^ ± 1.77
		7	6.03^b^ ± 0.19	6.57^a^ ± 0.57	6.71^a^ ± 0.21
		11.2	22.13^a^ ± 0.16	6.66^b^ ± 0.44	3.39^c^ ± 0.26
ovoalbumin	Hydrophilic (rinsed in water)	2.4	1.85^a^ ± 0.16	0.51^b^ ± 0.09	0.65^c^ ± 0.22
		7	14.54^a^ ± 0.08	1.86^b^ ± 0.36	0.00^c^ ± 0.00
		11.2	4.18^a^ ± 0.21	0.00^b^ ± 0.00	0.69^b^ ± 0.17
	Hydrophobic (rinsed in triton)	2.4	51.18^a^ ± 0.30	4.17^b^ ± 0.31	2.72^c^ ± 0.43
		7	23.71^a^ ± 0.09	3.00^b^ ± 0.49	2.178^c^ ± 0.36
		11.2	48.32^a^ ± 0.22	3.02^b^ ± 0.56	3.35^b^ ± 0.53

Key: n = 60 for each mean; various lowercase letters—the differences are statistically significant for comparisons in the rows for *p* ≤ 0.05 (ANOVAs and Duncan multiple range tests were performed for each pH within hydrophobic/hydrophilic proteins, separately); shadowed—proteolytic activities significantly decreased after varroacid treatment in comparison to the control. Protease activities were not observed in relation to casein and gelatine (data is not included here).

### 2.1. Cuticle Proteases

Hydrophobic protease activities were decreased and even not observed at all in relation to casein and gelatine (Table 1). Asparagine and serine proteases, but no thiolic proteases or metal-proteases, were detected on the apian cuticles because proteolytic activity was identified in the case of pepstatin‑A and phenylmethylsulfonyl fluoride (PMSF) but was not observed in relation to iodoacetamide or o-phenanthroline (diagnostic inhibitors of proteases).

Amitraz and oxalic acid treatments increased the number of acidic and alkaline protease (both hydrophilic and hydrophobic) and neutral hydrophobic protease bands, and reduced the number of bands associated with neutral hydrophilic proteases in electrophorograms (Table 2). Furthermore, the width of the bands of alkaline proteases increased, whereas that of neutral proteases diminished. Considering Table 1, it may be assumed that varroacide treatment may render cuticle proteins/peptides inactive. 

**Table 2:** SDS-PAGE zymography of the active proteases on *A. mellifera* worker cuticles.

		Control—no treatment				Amitraz treatment				Oxalic acid treatment			
		hydrophilic protein		hydrophobic protein		hydrophilic protein		hydrophobic protein		hydrophilic protein		hydrophobic protein	
pH	Rf (mm)	Band number	OD	Band number	OD	Band number	OD	Band number	OD	Band number	OD	Band number	OD
2.4	0–40	1	0.25	3	0.86	3	0.21	4	0.25	3	0.15	1	0.20
	41–60	1	0.40	2	0.35	1	0.26	3	0.43	1	0.35	1	0.23
	61–100	4	0.28	0	0	3	0.36	4	0.46	1	0.49	3	0.53
7.0	0–40	5	0.18	2	0.54	1	0.49	4	0.66	0	0	3	0.48
	41–60	2	0.20	0	0	1	0.63	1	0.92	2	0.47	1	0.74
	61–100	4	0.25	3	0.36	2	0.61	2	0.68	3	0.52	2	0.74
11.0	0–40	3	0.25	0	0	1	0.30	5	0.23	1	0.12	2	0.14
	41–60	0	0	0	0	1	0.23	1	0.15	1	0.19	1	0.20
	61–100	1	0.32	0	0	4	0.25	1	0.18	2	0.18	3	0.38

Key: Rf-—ranges of the protein migration path (0–40 high-molecular, 41–60 medium-molecular 61–100 low molecular); OD—width of the bands. The results of protease activities in SDS-PAGE zymography are the arithmetic mean (n = 60) of the results obtained in each group. A lack of any considerable differences between electrophoretic patterns within each group was observed even though the electrophoresis were performed five-times (the band pattern was very similar within each group) Therefore no statistics were applied.

### 2.2. Cuticle Protease Inhibitors

**Figure 2:**
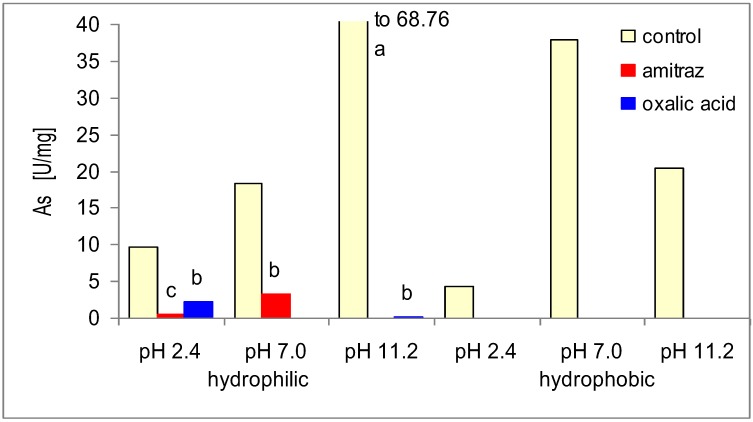
Mean (n = 60) activities of natural protease inhibitors (**As**) in the cuticle samples of *A. mellifera* workers treated with amitraz and oxalic acid in comparison to the control. Key: various lowercase letters—the differences are significant for *p* ≤ 0.05. Separate comparisons were made for each pH within hydrophilic and hydrophobic proteins (6 × ANOVAs and Duncan multiple range tests).

**Figure 3:**
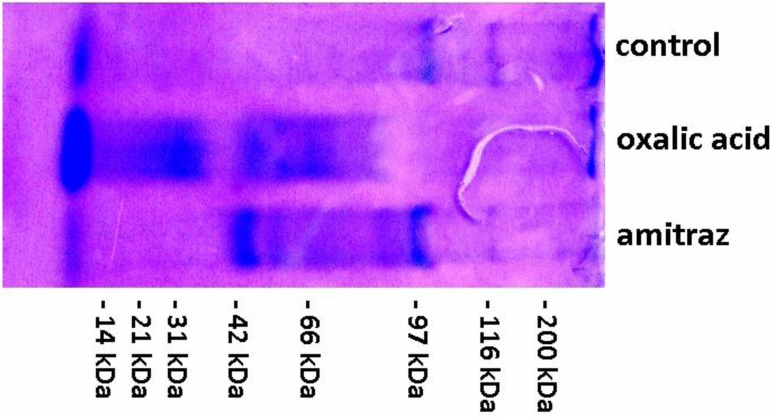
An electrophorogram (example) depicting serine protease inhibitor activities of the samples from cuticles of *A. mellifera* workers treated with amitraz and oxalic acid in comparison to the control.

Amitraz and oxalic acid caused a complete loss or considerable reduction (Figure 2) in the activities of natural inhibitors of acidic, neutral, and alkaline proteases (both hydrophilic and hydrophobic). In the electrophorograms of the control group, poorly pronounced narrow bands associated with both high- and low-molecular inhibitors of asparagine and serine proteases were observed (Figure 3, Table 3). After applying amitraz and oxalic acid, additional bands associated with high- and low-molecular protease inhibitors appeared. These were broader and well-pronounced at acidic and neutral but narrower and less prominent at alkaline pH. It may be assumed that amitraz and oxalic acid deactivated protease inhibitors or reacted with the proteases/substrates. Moreover, they may have destroyed the structure of one/multiple components of the protease/substrate/inhibitor complex. In all the cases more inactive cuticle proteins/peptides may appear.

**Table 3:** SDS-PAGE zymography of the active natural protease inhibitors on *A. mellifera* worker cuticles.

		Control—no treatment				Amitraz treatment				Oxalic acid treatment			
		hydrophilic protein		hydrophobic protein		hydrophilic protein		hydrophobic protein		hydrophilic protein		hydrophobic protein	
pH	Rf (mm)	Band number	OD	Band number	OD	Band number	OD	Band number	OD	Band number	OD	Band number	OD
2.4	0–40	4	0.21	0	0	5	0.73	2	0.74	4	0.65	5	0.63
	41–60	0	0	1	0.20	1	0.58	1	0.67	1	0.55	0	0
	61–100	3	0.21	4	0.14	5	0.53	4	0.56	2	0.58	3	0.61
7.0	0–40	2	0.16	1	0.23	2	0.23	4	0.35	3	0.26	1	0.23
	41–60	0	0	0	0	1	0.30	2	0.52	0	0	0	0
	61–100	0	0	1	0.21	1	0.27	4	0.34	2	0.26	5	0.39
11.0	0–40	3	0.37	4	0.23	2	0.14	2	0.16	2	0.08	0	0
	41–60	0	0	0	0	2	0.10	0	0	1	0.11	0	0
	61–100	3	0.36	0	0	0	0	3	0.28	1	0.04	3	0.23

Key: Rf—range of the protein migration path (0–40 high-molecular, 41–60 medium-molecular 61–100 low molecular), points of protease inhibitor bonding with proteases and substrates in the polyacrylamide gel; OD—width of the bands. The results of protease inhibitor activities in SDS-PAGE zymography are the arithmetic mean (n = 60) of the results obtained in each of the groups. A lack of any considerable differences between electrophoretic patterns within each group was observed even though the electrophoresis were performed five-times (the band pattern was very similar within each group). Therefore no statistics were applied.

### 2.3. Apian Cuticle Proteolytic System — General Comments

Our results showed that both amitraz and oxalic acid reduced the activities of the cuticle proteolytic systems of worker bees, especially as regards hydrophobic proteases. This was particularly noticeable in the case of natural protease inhibitors. This suppressive effect may stem from the varroacids’ toxicity. Howis *et al.* [23] proved that organic acids damage anatomic and physiological structures of the alimentary and body cover, and can facilitate pathogen penetration by debilitating the proteolytic system. Additionally, organic acids create a favorable environment for fungal development. Moreover, oxalic acid probably has a negative effect on the Krebs cycle. Drugs to fight *V. destructor* used to be selected by their efficacy, ease of application, and affordability [25]. In this context, amitraz seems a good solution. Nevertheless, our results reveal that amitraz is not advantageous as regards the proteolytic barrier. A “vicious circle” is created, in which drug use reduces resistance, leading to weaker colonies, and thereby to more losses in the apiary, which then require more intensive drug application. This finding corresponds with feedback from beekeepers, who have observed the spreading of various diseases in their hives after intensive use of the above drugs [26]. Amitraz-containing drugs considerably reduced the amount of the main biochemical compounds in the hemolymph of adult workers from their emergence until foraging age, and to a lower extent, the amount of proteins in the body tissues of the workers [27]. Our results demonstrate that amitraz also lowers protein concentration and suppresses proteolytic system activity on the body surface of honeybees. Therefore, among the side effects of therapy against *V. destructor*, we should enumerate the extenuation of the proteolytic barrier.

Our previous experiments proved that some external factors could supress the proteolytic barrier on the apian cuticle. In a polluted environment, lower values of protein concentrations, protease activities, and natural protease inhibitor activities were observed in the worker cuticles as compared with the cuticles of bees from an unpolluted environment [11]. Bee diet may also affect the barrier efficiency [28]. The present study revealed that amitraz and oxalic acid theraphy may also reduce cuticular proteolytic system activity and therefore may be regarded as harmful external factors.

### 2.4. Activity towards Model Microorganisms

The rinsings from the body surface of workers in the control group showed activity in relation to all the microorganisms (Table 4). In bees treated with amitraz and oxalic acid, no activity towards *A. niger* or *S. aureus* was observed. Moreover, after applying oxalic acid, no activity was identified in relation to *S. typhimurium* or *P. aeruginosa*. This may correspond with a low *in vitro* activity of the proteolytic system, due to the influence of those varroacids, and confirms their suppressive effect on the proteolytic barrier especially in the case of fungal infection. On the other hand, amitraz increased the activity towards *Bacillus subtilis*, *Salmonella typhimurium*, *Pseudomonas aeruginosa*. Hence, suppression was not always observed, though mostly.

**Table 4:** Antifungal and antibacterial activities of cuticle washings of *A. mellifera* workers treated with amitraz and oxalic acid, measured as the area of the infected medium on which there was no microorganism growth (**mm^2^**) in comparison to the control.

Group	Antifungal activity		Antibacterial activity			
	*Candida albicans*	*Aspergillus niger*	*Bacillus subtilis*	*Staphylococcus aureus*	*Salmonella typhimurium*	*Pseudomonas aeruginosa*
Control	978.57	23.36	78.36	308.61	126.49	101.33
Amitraz	212.50	0	756.51	0	259.62	207.24
Oxalic acid	340.69	0	154.18	0	0	0

Key: the portions/samples were polled (60 portions within each the group) and 1 polled sample per group was obtained. So, the totalized group effects were shown here and no statistics were applied.

Ants have protective microbial biofilms on their body surfaces that are affected by various chemical substances [29]. Grzywnowicz et al. [9] suspected that bees might also have such biofilms. If they do, amitraz and oxalic acid could suppress the biofilm activity (anti-pathogen cuticle barrier) conditioned by the proteolytic system. Recent research [30] shows that the V. destructor parasite is equipped with a set of enzymes that belong to the category of asparagine and cysteine proteases, as well as metal‑proteases with a pH range of only 2.0–6.5. In turn, as evidenced in this research, A. mellifera have asparginic and serine proteases on their body surface whose pH range is much broader (2.4–11.2) than that of the acarids, which could help bees block “foreign” proteins. 

## 3. Experimental Section

### 3.1. The Experimental Design

Three experimental groups with 5 colonies of Buckfast bees in each (headed by sister-queens) were monitored (15 colonies). In the first group bees were sprayed with a 3.2% oxalic acid solution, 50 mL per colony. The solution was made using 15 g oxalic acid dihydrate, 200 g sucrose, and 200 mL water [24]. Each colony in the second group was fumigated with one tablet of Apiowarol AS^®^ (12.5 mg amitraz) single-time according to the producer’s recommendations. The treatments were repeated in 2009 and 2010 at the end of July. In the third, control group, neither amitraz nor oxalic acid treatments were performed.

### 3.2. Sampling and the Data Base

Three samples comprising 10 nest worker bees were collected from each colony just before and two weeks after the treatments consecutively in 2009 and 2010 (together 6 samples). Totally the procedure produced 5 colonies × 6 samples × 2 years = 60 samples = 600 bees for each of the three group. The material was frozen in sterile bags at −8°C and stored for 1–2 months. Then, the samples were refrozen and rinsed in 10 ml distilled water for 20 seconds to remove impurities. Proteins were not found in the rinsings using the Lowry method as modified by Schacterle and Pollack [31]. Therefore, the rinsings were discarded. Subsequently, the samples were shaken/rinsed for 4 min at 3,400 rpm in 10 mL distilled water, and finally, after filtering through Miracloth, a solution was obtained that mostly contained hydrophilic proteins. The solution obtained from each sample was then divided into 4 portions, poured into four Eppendorf tubes, and frozen again at −40°C. The procedure produced:

2 mL—for determining protease and protease inhibitor activities (portion a),
2 mL—for electrophoretic assays (portion b),
2 mL—for determining antifungal and antibacterial activities *in vivo* (portion c),
2 mL—reserve (portion d).

Altogether, we obtained 60 samples × 4 portions = 240 portions per group.

Then, the solid samples that remained on the Miracloth were again shaken/rinsed (4 min at 3,400 rpm) in a 1% detergent solution (Triton X-100) in distilled water (10 mL). Similarly to the first rinsing, 4 portions (2 mL) were created from each sample, now containing mostly hydrophobic proteins.

The entire procedure resulted in a total of 1440 portions (3 groups × 60 samples × 2 rinsings × 4 portions).

### 3.3. The Analytical Procedures

For each of the three groups, 62 µL of the solution were sampled from each “**portion a**”, and mixed to create a one pooled group-sample (3 pooled group-samples, each consisting of 60 samples) to determine optimal pH values, at which protein activities were high. The values amounted to 2.2–3.4, 6.4–7.6, and 8.2–11.6 in the pooled samples. Therefore, the activities of acid, neutral and alkaline proteases/protease inhibitors were decided to be determinate at pH 2.4, 7.0 and 11.2, respectively. 

Next, the remainder (1.938 mL) of each “**portion a**” was analyzed (portions were not pooled in this case; n = 60) as follows:

- the general protein content by the Lowry method, as modified by Schacterle and Pollack [31];
- the proteolytic activity in relation to the substrates (gelatine, hemoglobin, ovoalbumin, albumin, cytochrome C, casein were chosen on the basis of our former studies [9,10,11]), according to the Anson method [32]
- the proteolytic activity in relation to the diagnostic inhibitors of proteolytic enzymes (pepstatin A, PMSF, iodoacetamide, o-phenanthroline), by the Lee and Lin method [33];
- the activities of acidic, neutral, and alkaline proteases according to the Anson method [32];
- the levels of natural inhibitors of acidic, neutral, and alkaline proteases, based on the Lee and Lin method [33].

Polyacrylamide gel electrophoresis was performed using “**portions b**”, which had been previously lyophilized and then combined with 100 µL distilled water. Each portion (n = 60) was analyzed separately in this case. The following analyses were conducted:

- protease detection using the Laemmli method [34]; *i.e.*, SDS-PAGE method.
- detection of inhibitors of asparagine and serine proteases using the Felicioli *et al.* method [35]; *i.e.*, SDS-PAGE method.

### 3.4. *In Vivo* Laboratory Tests

“**Portions c**” from each group were pooled separately (3 pooled group-samples; each consisting of 60 portions), and then lyophilized to determine antifungal and antibacterial activities. To this end, the lyophilizates were combined with 200 µL distilled water. Subsequently, 10 µL of the mixtures were spackled on culture media using the double application method, with:

- SABG [36]—to determine activity in relation to *Aspergillus niger*,
- YPD [37]—to determine activity in relation to *Candida albicans*,
- LB [38]—to determine activity in relation to *Staphylococcus ureus* (ATCC 25923), *Bacillus subtilis* (ATCC 6633), *Micrococcus luteus* (ATCC 7468), *Salmonella typhimurium* (ATCC 13311), *Pseudomonas aeruginosa* (ATCC 17853), and *Escherichia coli* (ATCC 10536).

In all microbiological tests, each dish was photographed (SONY α100) to determine the area on which there was no microorganism growth, using the MultiScanBase [39].

### 3.5. Statistics

To statistically verify an influence of amitraz and oxalic acid (experimental factors) on the protein contents as well as on the protease and protease inhibitor activities, one-way ANOVAs and the Duncan multiple range tests were performed using the SAS [40].

## 4. Conclusion

Amitraz and oxalic acid may upset the cuticle proteolytic defense system in *A. mellifera*. Therefore, a Varroa treatment including these drugs may make honeybee workers more susceptible to pathogenic (especially fungal) invasions. The bees were not age-controlled, thus the drugs’ effects might be direct and indirect (via pupae and within-colony environment; compare [41]).
